# Preservation of Antigen-Specific Functions of αβ T Cells and B Cells Removed from Hematopoietic Stem Cell Transplants Suggests Their Use As an Alternative Cell Source for Advanced Manipulation and Adoptive Immunotherapy

**DOI:** 10.3389/fimmu.2017.00332

**Published:** 2017-03-23

**Authors:** Giuseppina Li Pira, Stefano Di Cecca, Simone Biagini, Elia Girolami, Elisabetta Cicchetti, Valentina Bertaina, Concetta Quintarelli, Ignazio Caruana, Barbarella Lucarelli, Pietro Merli, Daria Pagliara, Letizia Pomponia Brescia, Alice Bertaina, Mauro Montanari, Franco Locatelli

**Affiliations:** ^1^Department of Pediatric Hematology and Oncology, IRCCS Bambino Gesù Children’s Hospital, Rome, Italy; ^2^Department of “Medicina Clinica e Chirurgia”, University of Naples Federico II, Naples, Italy; ^3^Department of Pediatrics, University of Pavia, Pavia, Italy

**Keywords:** HLA-haploidentical transplantation, graft manipulation, αβ T cells, alloreactivity, antigen-induced activation, B-cell presentation

## Abstract

Hematopoietic stem cell transplantation is standard therapy for numerous hematological diseases. The use of haploidentical donors, sharing half of the HLA alleles with the recipient, has facilitated the use of this procedure as patients can rely on availability of a haploidentical donor within their family. Since HLA disparity increases the risk of graft-versus-host disease, T-cell depletion has been used to remove alloreactive lymphocytes from the graft. Selective removal of αβ T cells, which encompass the alloreactive repertoire, combined with removal of B cells to prevent EBV-related lymphoproliferative disease, proved safe and effective in clinical studies. Depleted αβ T cells and B cells are generally discarded as by-products. Considering the possible use of donor T cells for donor lymphocyte infusions or for generation of pathogen-specific T cells as mediators of graft-versus-infection effect, we tested whether cells in the discarded fractions were functionally intact. Response to alloantigens and to viral antigens comparable to that of unmanipulated cells indicated a functional integrity of αβ T cells, in spite of the manipulation used for their depletion. Furthermore, B cells proved to be efficient antigen-presenting cells, indicating that antigen uptake, processing, and presentation were fully preserved. Therefore, we propose that separated αβ T lymphocytes could be employed for obtaining pathogen-specific T cells, applying available methods for positive selection, which eventually leads to indirect allodepletion. In addition, these functional T cells could undergo additional manipulation, such as direct allodepletion or genetic modification.

## Introduction

HLA-haploidentical hematopoietic stem cell transplantation (haplo-HSCT) from a related donor is a suitable option for patients affected by many malignant and non-malignant hematological diseases (1–3). In fact, patients have a high chance of having a haploidentical related donor within their family group, and pediatric patients, in particular, can rely on both parents as potential donors. Nevertheless, due to the incompatible haplotype, the risk of graft-versus-host disease (GvHD) is high in haplo-HSCT (2). Removal of T cells encompassing the alloreactive precursors has been applied to prevent GvHD (4, 5), as recently reviewed (6). This procedure is effective in preventing GvHD, but a concomitant increase of graft rejection (7, 8), loss of T-cell mediated graft-versus-leukemia (GvL) effect, with higher incidence of relapse of the primary malignant disease (9–12), and loss of graft-versus-infection (GvI) effect, with a higher risk of severe opportunistic infections due to delayed reconstitution of pathogen-specific T-cell immunity (13–15), have been also observed. In comparison to a bone marrow-derived graft, the introduction of G-CSF-mobilized HSC (16) made the apheretic product a more convenient cell source for haplo-HSCT. Recently, more selective procedures of graft manipulation have been introduced to remove alloreactive precursors within well-defined T-cell subsets, preserving other mononuclear cells that exert protective immune functions. Selective T-cell depletion can include (i) removal of αβ T cells, which encompass the alloreactive precursors, sparing γδ T cells, and natural killer (NK) cells, which play a protective role against opportunistic infection and regrowth of leukemia cells (17) and (ii) removal of the CD45RA *naïve* T-cell fraction containing alloreactive precursors, sparing the memory fraction containing T cells responsive to opportunistic pathogens (18–20). The first procedure, based on removal of αβ T cells with anti αβ TCR antibodies bound on paramagnetic microbeads, which are retained by a magnetic column, is now commercially available with certified reagents, protocols, and automated instrumentation (Miltenyi Biotec, Bergish Gladbach, Germany). This procedure includes concomitant removal of B cells with anti-CD19 antibodies with the purpose of reducing the risk of EBV-associated posttransplant lymphoproliferative disease. Clinical results demonstrating the safety and efficacy of this procedure have been recently reported (21–23).

The αβ T-cell and B-cell depleted product (graft) contains, in addition to CD34 HSC, other mononuclear cells such as NK cells, γδ T cells, and monocytes/dendritic cells (MoDC), which exert positive immune functions (21). The labeled αβ T cells and B cells retained by the magnetic column represent the non-target (NT) population. If the magnetic field is withdrawn from the paramagnetic column, the retained cells can be eluted and collected as the NT fraction, but they are generally disposed of.

We considered the NT fractions as an immunological asset worth analyzing for specific functions after the *in vitro* graft manipulation. NT cells, in fact, could be considered as an alternative source of T cells for unmanipulated donor lymphocyte infusion (DLI) to control/prevent infectious complications (GvI effect) or to prevent/treat relapse of the primary malignancy (GvL effect) (24–27). Additionally, NT cells can be a valuable starting material to obtain antigen-specific T cells able to accelerate immune reconstitution (28), by using direct selection procedures based on multimer technology as described on recent reports (29–31). These reports are of special relevance in this context as they demonstrate that low doses of selected T cells effectively provided a GvI effect and could expand *in vivo* to reconstitute a protective T-cell response. Furthermore, the same T cells can be considered for further advanced manipulation (32–35) for the introduction of suicide genes or for expression of novel engineered T-cell receptors.

In light of these considerations, in this work we tested the preservation of antigen-specific functions of αβ T cells and the antigen-presenting function of B cells present in the NT fraction after the depletion procedures.

## Materials and Methods

### Reagents and Media

The kit for αβ T-cell/B-cell depletion includes reagents and disposable bags with interconnecting tubing in addition to the in-line magnetic column (Miltenyi, Bergish Gladbach, Germany). The PBS-EDTA buffer (Miltenyi) was supplemented with human serum albumin (HSA, Grifols, Barcelona, Spain). Ficoll (Lymphoprep) was obtained from Sigma (St. Louis, MO, USA). RPMI 1640 with HEPES buffer, l-glutamine, and antibiotics were from Euroclone (Milan, Italy). PPD was purchased from Statens Seruminstitut (Copenhagen, Denmark). CMV, EBV, and adenovirus antigens were obtained from Microbix Biosystems (Mississauga, ON, Canada) as lysates of infected cells centrifuged to remove cell debris. The CMV pp65 peptide library (pepmix, 15mer peptides overlapping by 11 residues) was purchased from JPT (Berlin, Germany). Reagents for IFNγ ELISA were from Mabtech (Stockholm, Sweden) and ^3^H-thymidine (specific activity 0.25 TBq/mmole) was from Perkin Elmer (Boston, MA, USA). Monoclonal antibodies (Moab) for cell phenotyping were from Becton Dickinson (San Josè, CA, USA), and they were used in combinations previously described (36).

### Collection of NT Cells

Donors of cells for haplo-HSCT underwent an apheretic session after HSC mobilization with G-CSF (22, 36). The cells in the apheresis (Aph) bag were processed to remove αβ T cells and B cells, as described in Ref. (37), following the detailed protocol provided by Miltenyi. Briefly, cells in the Aph bag were tagged with biotin-conjugated anti-αβ TCR Moab. After incubation and washing, the cells were labeled with paramagnetic beads conjugated to anti-biotin and anti-CD19 antibodies. After washing to remove unbound beads, the cell suspension was loaded on the CliniMACS device to automatically process cells through a magnetic column, which retains the labeled cells (αβ T cells and CD19 B cells). The effluent cells in the collection bag (CB) included CD34 HSC, hematopoietic precursors, γδ T cells, NK cells, MoDC, and represented the graft ready for infusion into the recipients. The average performance of 200 procedures, evaluated as depletion efficiency of unwanted cells and as recovery efficiency of desired cells (38), matched the data from other reports (22, 36, 37, 39). The retained population, consisting of αβ T cells and B cells, was designated as the NT fraction. This fraction could be eluted from the column upon withdrawal of the magnetic field. Samples from the different fractions obtained from 20 depletion procedures performed on 20 donors were saved for the experiments described here. HLA typing and serostatus of the donors is reported in Table 1. Positive serostatus was defined according to thresholds provided by the clinical laboratory.

**Table 1:** **HLA typing and viral serological status of the 20 donors included in the study**.

Donor #	HLA typing				Serology		
	A	B	C	DR	CMV	EBV	AdV
1	30, 1	35, 37	4, 6	4, 15	+	+	+
2	31, 11	35, 51	4, 7	1	+	+	+
3	23, 24	49, 27	7, 15	1, 11	+	+	+
4	3	35, 53	15, 4	8, 13	+	+	−
5	24, 1	51, 37	16, 6	13, 11	+	−	+
6	24, 2	44, 27	7, 2	4, 3	+	+	+
7	2, 32	44	7, 5	4, 4	+	+	+
8	30, 11	13, 15	6, 4	7, 13	+	−	−
9	2, 29	13, 39	4, 6	4, 4	+	+	+
10	2, 26	14, 55	3, 8	1, 13	+	−	+
11	11, 3	18, 14	7, 8	14, 13	+	+	nt
12	2	39, 13	12, 6	16, 7	+	+	+
13	1, 31	44, 51	7, 15	7, 4	+	+	+
14	24, 31	44, 51	2, 15	4, 15	+	+	+
15	1, 2	8, 57	7	3, 7	+	+	+
16	24, 11	35, 15	4, 1	11, 7	+	+	+
17	30, 23	14, 7	8, 7	10, 11	+	−	+
18	1, 68	35, 18	7, 7	1, 11	+	−	+
19	22, 23	38, 41	12, 17	13, 7	+	+	+
20	11, 2	35, 51	4, 15	4, 4	−	+	+

*Positive serological status was defined according to thresholds provided by the clinical laboratory*.

### Processing of NT Cells

Samples were obtained from the Aph bag, from the CB, and from the NT fraction. Cells were used for phenotypic characterization to evaluate procedure efficiency and for testing functional activity, as described here. Cells were kept in PBS–EDTA–HSA 0.5% buffer at 4°C for up to 24 h before processing. After washing with PBS, cells were loaded on conventional Ficoll gradients to obtain the mononuclear fraction. This step was not required for NT fractions, which already contained purified αβ T cells and B cells, but at any rate it was used for better parallel comparison with the other cell fractions. Ficoll passage and washing of the NT fractions removed the residual free beads that were eluted from the column along with labeled cells. Cells were resuspended at 2 × 10^6^ cells/ml in RPMI 1640 medium supplemented with l-glutamine 2 mM, 2-mercapto-ethanol 5 × 10^−5^ M, penicillin 100 U/ml, and streptomycin 100 μg/ml. Heat-inactivated human AB serum (Sigma) was added at 5% final concentration to produce the complete medium. Average cell viability was >90%.

### Functional Assays for Antigen-Specific Responses

Allospecific responses against T-cell depleted allogeneic PBMC from random donors as stimulator cells were tested in one-way mixed lymphocyte reactions (MLR). T-cell depletion was performed using anti-CD3 magnetic beads (Dynal, Thermo Fisher) following the manufacturer’s instructions. T-depleted stimulator cells from four subjects were pooled and frozen in aliquots as an allo-pool in order to maximize responder versus stimulator HLA disparity. When needed, cells were thawed, washed, and irradiated (30 Gy) before use. Responder cells were seeded at 100 μl/well (2 × 10^5^ cells) with an equal number of stimulator cells in 100 μl in 96-well, flat bottom microtiter plates. Responses were tested as IFNγ secretion after 2-day incubation. ELISA assays were run on culture supernatants following manufacturer’s instructions. Results are given as picogram per milliliters IFNγ or as stimulation indexes (SI, picogram per milliliters with antigen: picogram per milliliters background). A positive alloresponse was defined as having an SI ≥ 10. Responses to soluble antigens were tested on 2 × 10^5^ cells in 200 μl medium. Specific antigens included CMV, EBV, adenovirus lysates, CMV pp65 peptide mix, and PPD. Antigens were used at 5 μg/ml final concentration. After 5-day incubation, cultures were pulsed with 18 kBq ^3^H-thymidine for 18 h and harvested on glass fiber filters. Dry filters were counted on a Microbeta Trilux 1450 counter (Wallac, Perkin Elmer). Results are expressed as SI (cpm with antigen/cpm background). An SI ≥ 3 defined an antigen-specific positive response.

Statistical analyses were performed using the Graph Pad Prism 6.1 version software. Significant differences between sets of data were determined by the Mann–Whitney test. Correlation between paired data sets was determined using the Pearson’s coefficient. Highly significant differences were defined by *p* < 0.01.

## Results

### Depletion Efficiency and Enrichment of αβ T and B Cells

**Figure 1:**
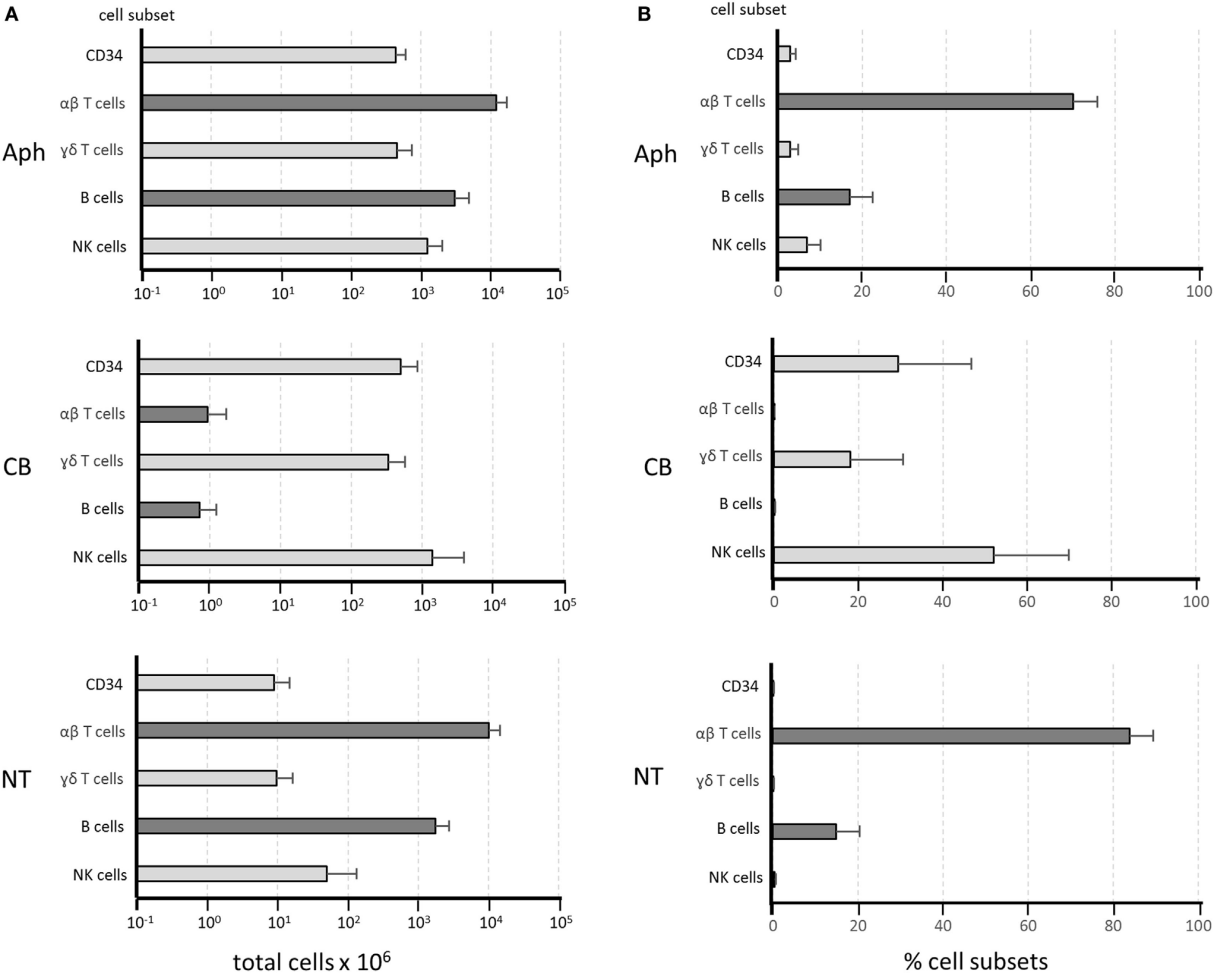
**Composition of cellular fractions obtained from αβ T-cell/B-cell depletion procedures**. Twenty depletion procedures were used for the reported experiments. Samples of the different cell fractions—apheresis (Aph), collection bag (CB), and non-target (NT) fraction—were evaluated for cellular composition. Dark gray bars indicate αβ T cells and B cells, while light gray bars refer to the other subsets. Results are shown as mean values ± SD of total cell numbers for each subset on a log scale in panel **(A)**. Relative frequency of the same subsets is shown in panel **(B)** as percentages of the total NC identified by flow cytometry. The Mann–Whitney test was used to define non-significant differences in αβ T-cell and B-cell numbers **(A)** or frequencies **(B)** between Aph and NT samples, indicating full recovery of the depleted cells in the NT fractions. By contrast, highly significant depletion for αβ T cells and B cells can be observed in panels **(A,B)** by comparing the Aph and the CB samples.

Figure 1 shows the composition of the manipulated cell fractions in 20 different procedures from which samples were obtained for this study. Most CD34 HSC, NK cells, and γδ T cells were recovered in the CB fractions. A remarkable depletion was achieved for αβ T cells and B cells, with mean values of around 12,000 × 10^6^ and 3,000 × 10^6^ in the Aph samples versus 1 × 10^6^ and 0.8 × 10^6^ in the CB fractions. These cell subsets were recovered in the NT fractions, with mean values of around 10,000 × 10^6^ and 2,800 × 10^6^, corresponding to a recovery efficiency of 84 and 93%, respectively. Since these figures obtained from 20 procedures overlap with the results obtained from over 200 procedures performed in our laboratory (38), the data reported below can be considered fully representative of the fractions obtained with the standard depletion procedure. In order to better visualize the αβ T- and B-cell depletion from the CB fractions and their recovery in the NT fractions, the graphs in Figure 1B express the results as relative percentages.

### Preservation of T-Cell Alloreactive Responses

**Figure 2:**
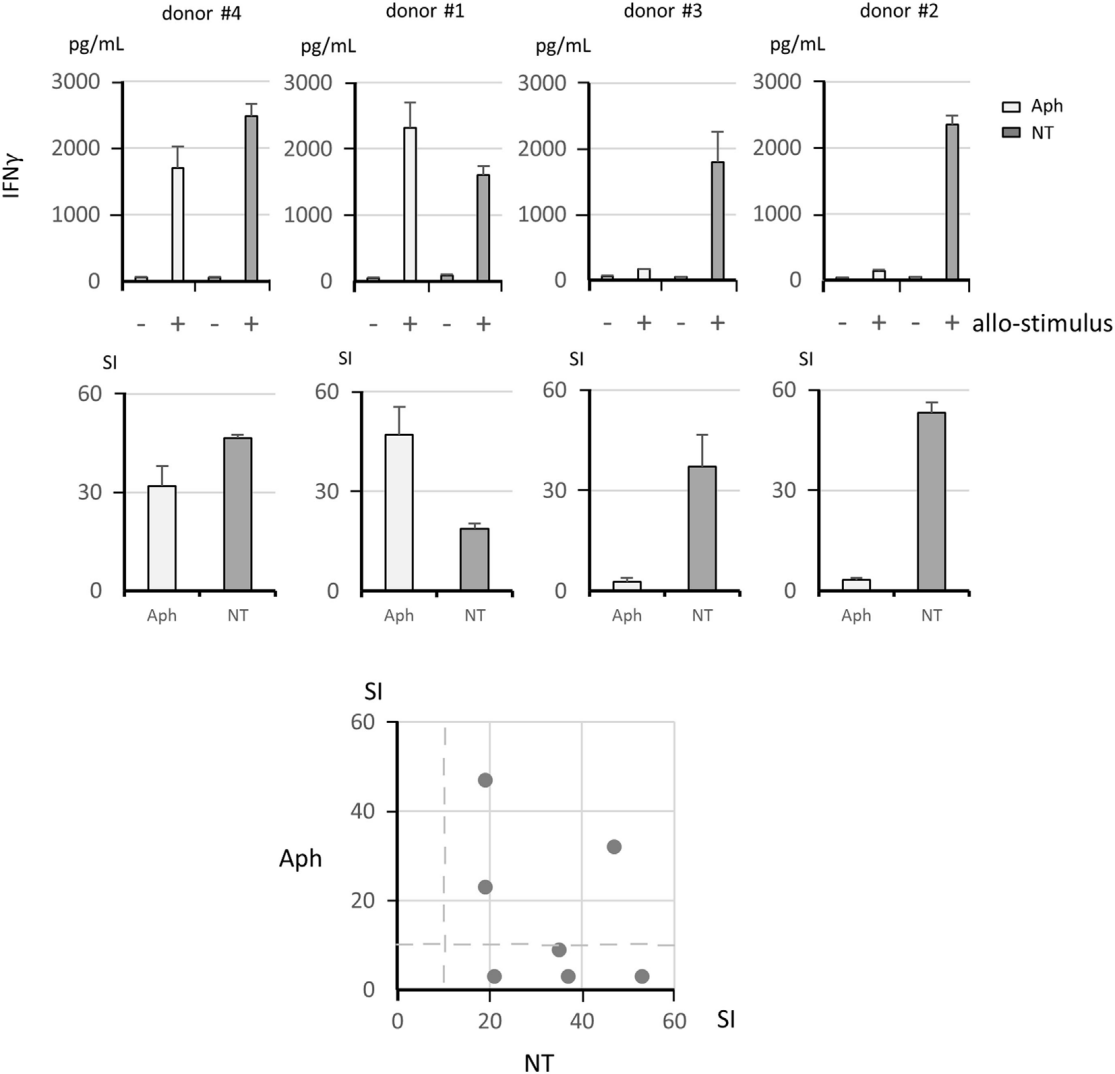
**Analysis of alloreactive responses**. The upper panels show IFNγ concentration as picogram per milliliters measured by ELISA on culture supernatants from mixed lymphocyte reaction assays (+, allogeneic stimulator cell pool; −, autologous control cells). The IFNγ concentrations have been normalized and referred to 10^5^ αβ T cells for each donor, considering that these cells were present at different concentrations in the Aph and in the non-target (NT) fractions. The middle panels show the corresponding stimulation indexes (SI). Four representative donors are shown, with vigorous responses in both Aph and NT samples in the two left hand panels and vigorous responses in the NT fractions only in the two right hand panels. The bottom panel shows a summary plot of SI for Aph and NT samples for each donor, with no statistically significant correlation between the paired data (Pearson’s coefficient test). Dashed lines indicate SI = 10.

Representative examples of MLR are shown in Figure 2. Alloreactive responses were tested in Aph samples and in NT samples. Upper panels show results as IFNγ concentration, while middle panels show results as SI. It should be noted that IFNγ concentrations have been normalized and referred to 10^5^ αβ T cells for each donor to take into account the fact that different numbers of αβ T cells were present in the Aph and in the NT fractions. The two graphs on the left are examples of concordant responses, with high alloreactivity in both Aph and NT cells. The two graphs on the right provide examples of discordant responses. The bottom plot shows responses of Aph and of NT cells for each tested donor. Assuming a threshold of SI ≥ 10 for a positive alloresponse, 7/7 NT fractions were positive, while 3/7 Aph samples were positive. No opposite cases of NT negative and Aph positive alloresponses were observed. Overall correlation between alloresponses in NT and in Aph fractions was not statistically significant.

### Maintenance of Specific T-Cell Response to Protein Antigens

**Figure 3:**
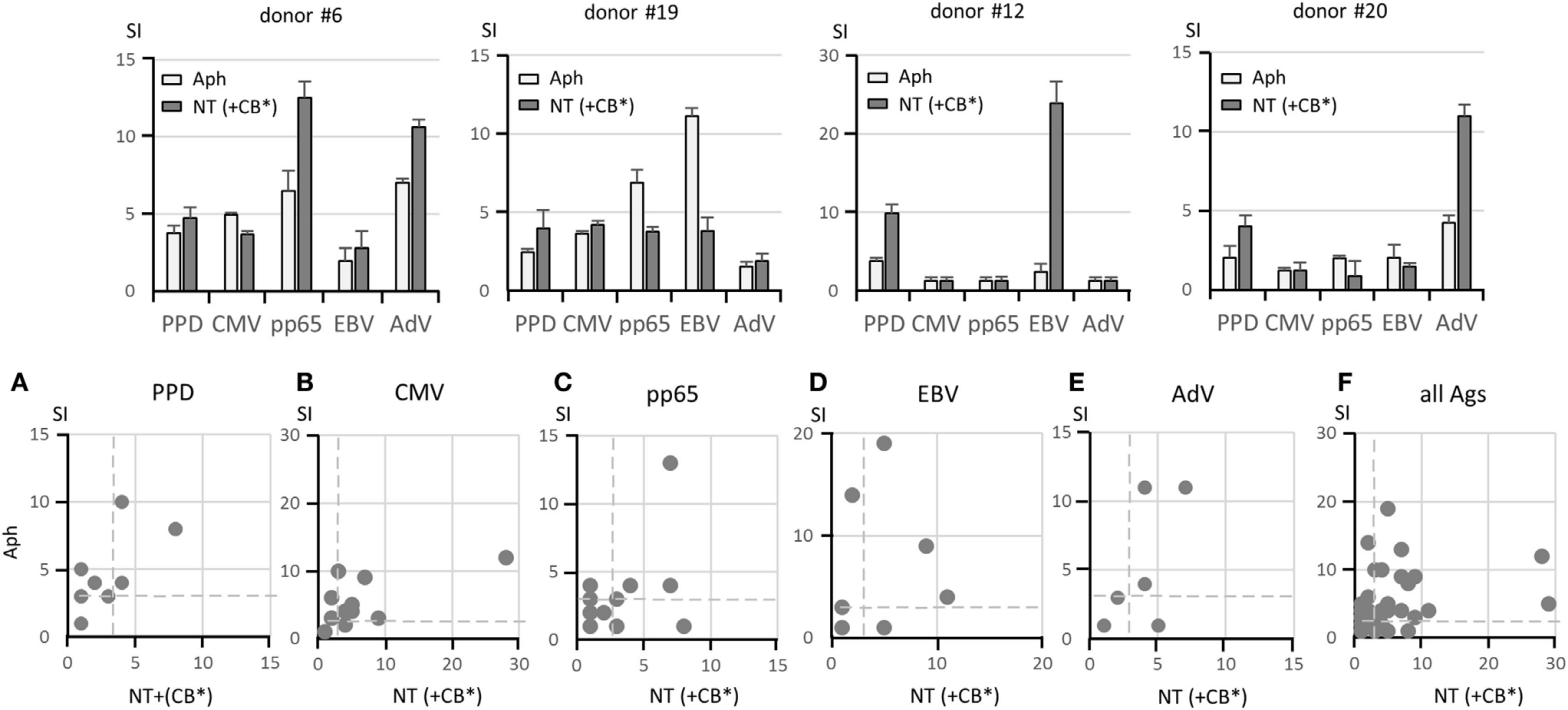
**Responses to soluble antigens**. The upper panels show representative lymphoproliferation assays of Aph and non-target (NT) (+irradiated CB*) samples from four donors stimulated with the following antigens: PPD, CMV lysate, CMV pp65 pepmix, EBV lysate, and AdV lysate. Different response profiles are shown. Responses to PPD, CMV lysate, CMV pp65 pepmix, EBV lysate, and AdV lysate in 20 pairs of Aph and NT (+CB*) samples are shown in the lower panels **(A–E)** as dot plots. A comprehensive view of responses to all antigens is given in panel **(F)**. Results are shown as stimulation indexes (SI). Dashed lines indicate SI = 3. Responses with identical SI in the two fractions overlap in the same position, and thus fewer than 20 dots may appear. A statistically significant correlation could not be established between Aph and NT (+CB*) responses (Pearson’s coefficient test).

Since we did not know whether B cells in the NT fractions had reduced APC function due to a possible interference with this function by anti-CD19 beads bound to the cell surface or because of extensive manipulation, we added irradiated cells from the CB fractions (CB*), which contain professional APC represented by MoDC, to ensure an adequate APC function. Four representative examples of antigen-specific responses expressed as SI are shown in Figure 3, upper panels. Lower panels (Figures 3A–E) illustrate the response for each antigen in the Aph fractions and in the NT (+CB*) fractions for all donors. A positive response to one or more antigens seen in NT fractions suggested that αβ T cells were not impaired in their specific capacity to recognize antigens. A summary of responses to the various antigens is shown in Figure 3F. A possible association between serostatus and T-cell response to viral antigens was considered by defining the frequency of concordant responses, either positive or negative. The seropositive status defined using the threshold provided the clinical laboratory and the positive T-cell response was defined by SI ≥ 3 in Aph and/or NT fractions. Concordance was 84% for CMV responses, 89% for EBV responses, and 47% for AdV responses. This suggests that T-cell responses should be defined independently of the donor’s serostatus.

### Ability of B Cells to Process and Present Protein and Peptide Antigens to Specific T Cells

**Figure 4:**
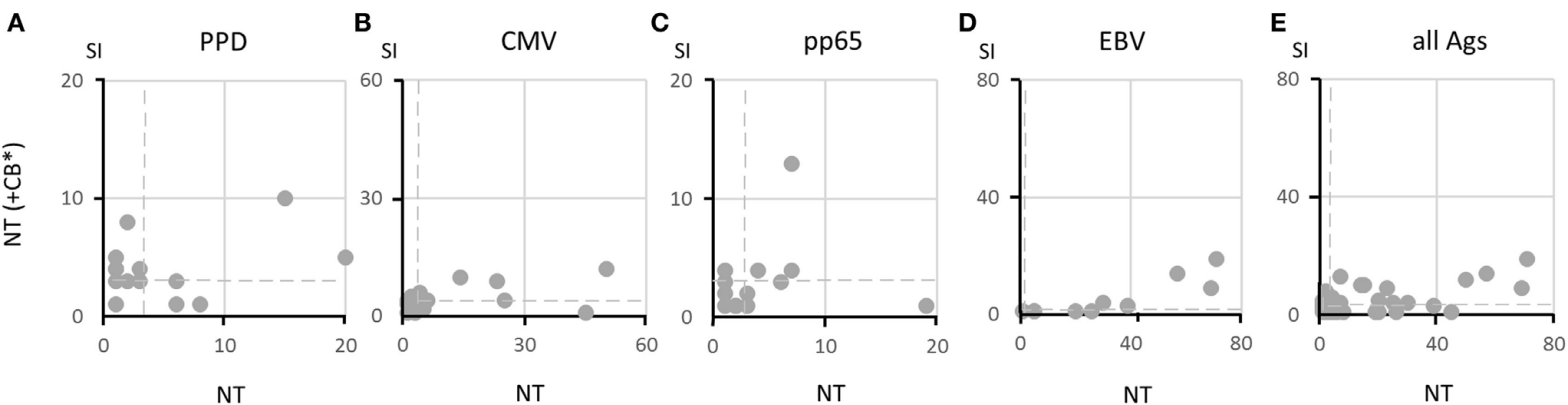
**Ability of B cells to present soluble antigens to specific T cells**. Responses to various antigens by 20 pairs of non-target (NT) (+irradiated CB*) and NT samples are shown in panels **(A–D)** as dot plots. Responses with identical stimulation indexes (SI) in the two fractions overlap in the same position, and thus fewer than 20 dots may appear. Dashed lines indicate SI = 3. Responses to all antigens are summarized in panel **(E)**, showing that responses by NT cells are more vigorous than responses by NT (+CB*) cells. A statistically significant correlation could not be established between Aph and NT (+CB*) responses (Pearson’s coefficient test).

After demonstrating that αβ T cells in the NT fractions are functionally intact, we evaluated whether B cells present in the same fractions were capable of exerting their APC function or whether additional professional APC, such MoDC present in the CB fractions, were necessary for optimal T-cell activation. By using protein antigens, such as viral lysates in addition to the CMV pp65 peptide mixture, it was possible to test the antigen uptake and processing ability and not simply the APC function of B cells. Figure 4 demonstrates that MoDC in the CB fractions irradiated at 30 Gy, which abolishes the function of residual T cells but preserves the function of APC, were of no advantage when added as supplementary APC and, on certain occasions, were even detrimental to specific T-cell responses. This is shown in Figures 4A–D, in which all data are reported for each antigen. It appears that B cells contained in the NT fractions were fully functional as APC and required no additional support. The overall superior responsiveness of NT cells alone is evident also from Figure 4E, in which SI for all antigens have been plotted.

### Comparison of Response in Aph and in NT Samples

**Figure 5:**
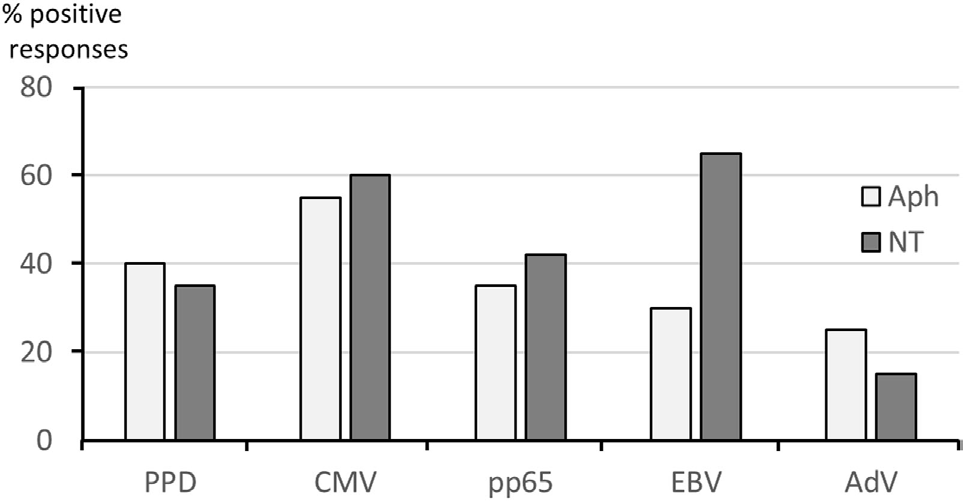
**Frequency of antigen-specific responses in paired samples**. Positive responses to soluble antigens defined by stimulation indexes (SI) ≥3 are shown by bars referring to Aph (light gray) and to non-target (NT) (dark gray) samples.

For a better evaluation of the discrepancies between NT and Aph responses observed on several occasions, Figure 5 shows the percent positive responses defined by an SI ≥ 3 to each tested antigen in all Aph samples and NT samples. The highest frequency of positive responses in Aph and/or NT samples was seen with CMV (55% for Aph and 60% for NT), while the lowest frequency was observed for AdV (24% for Aph and 16% for NT).

## Discussion

Recovery of antigen-specific T cells from G-CSF mobilized aphereses has been the subject of some conflicting reports. While G-CSF can increase the number of circulating lymphocytes (40–42), the observation that G-CSF inhibits the production of Th1-type cytokines raised concerns on the use of mobilized T cells with respect to immune reconstitution (43–47). Nevertheless, T cells from mobilized donors proved suitable for specific selection using either multimers (48, 49) or activation markers for enrichment and expansion (50, 51), with effectiveness comparable to that of T cells from non-mobilized samples.

In the perspective of selecting virus-specific T cells from NT fractions, we need to discuss several aspects related to the isolation procedure. Basically, two approaches are to be considered, one based on multimer selection and one based on selection with the cytokine secretion system (CSS).

In the first case, the multimer technology, initially described in Ref. (52), was successfully applied for selection and reinfusion of virus-specific T cells (53, 54). Nevertheless, the introduction of reversible streptamers, which can be eluted from the selected T cells (55), allowed more studies to demonstrate that T cells isolated with these reagents are functionally superior to CTL lines with the same CMV specificity in terms of protective potential (56), provide better protection when expressing high avidity TCR (57), have a remarkable proliferative capacity after infusion (29), can be serially transferred between experimental animals thanks to their stemness (30), and do not need to be specific for immunodominant epitopes (58). Finally, specific T cells can be isolated with minimal manipulation as cellular products for clinical use (31). Therefore, streptamer-enriched T cells can be proposed for immune reconstitution (59), as well as for immunotherapy of selected tumors (59, 60). A variation on the theme of reversible streptamers was introduced to dissociate multimers from CMV-specific selected cells by coupling onto cobalt-based magnetic beads histidine-tagged MHC molecules, which monomerize in the presence of histidine as specific inhibitor (61).

In the second case, the CSS, described in Ref. (62, 63), was applied for selection of CMV-specific T cells in *in vitro* studies (64, 65). *In vivo* studies demonstrated successful immune reconstitution with T cells specific for CMV (66), adenovirus (67), and EBV (68, 69).

Both these approaches are appealing as they allow positive selection of specific and functional T cells in a period of time much shorter than that needed to generate after rounds of antigen stimulation-specific T-cell lines, as it has been used in the past (70–72). We should keep in mind that multimers allow selection to be performed in a few hours with a simple procedure, without the requirement of an activation step, but they can be used only with selected epitopes on a limited array of HLA alleles and mostly for selection of CD8 T cells. In contrast, the CSS procedure requires an activation phase and more complex steps of cell processing, but it can activate both CD4 and CD8 cells simultaneously, is not limited by the available recombinant HLA alleles, and mixtures of peptides from different proteins and viruses can be used.

The use of multimer-selected CMV-specific T cells for immune reconstitution has been described in the past (53, 54), proving to be a rapid and highly effective procedure. This approach has been recently reviewed and advocated (73), but some limitations, intrinsic in the multimer strategy, should be considered. Multimer selection for immune reconstitution is limited to CD8 CTL and restricted to the HLA alleles available at present for multimer construction. Immunodominant epitopes must be known for each pathogen, and different multimers must be constructed with each peptide and each HLA allele. These aspects also apply to multimer-based selection of specific T cells from G-CSF mobilized aphereses (48). In addition, multimer-selected T cells from mobilized samples showed a reduction of antiviral function when compared to non-mobilized products (49).

Several conclusions can be drawn from the experiments presented in our study. First, antigen-specific responses by αβ T cells in the NT fractions were well preserved, in spite of manipulation steps, which included labeling, washing, column passage, and elution. In several cases, responses by NT cells were even higher than those obtained with unprocessed T cells from the Aph fractions. Second, antigen processing and presenting capacity was exerted by B cells in full, as additional APC, such as MoDC from the CB population, did not increase specific T-cell responses. The preserved T-cell function indicates that anti-αβ TCR–bead complexes on T cells did not interfere with cell functions (antigen recognition, proliferation, and cytokine secretion), as demonstrated by vigorous responses of NT cells in comparison with unmanipulated Aph cells. To explain this observation, we propose that only a fraction of TCR molecules are engaged by anti αβ TCR Mab on the cell surface or that antibody–bead complexes are rapidly internalized after cell incubation in culture (Miltenyi, personal communication). Similar to the T-cell hypothesis, anti-CD19–bead complexes on B cells, even if persisting on the cell surface, did not interfere with antigen uptake, processing, and presentation, suggesting that either engagement of CD19 does not affect the APC function or not all CD19 molecules are bound or complexes are promptly recycled from the B cell surface as an active process during cell incubation.

With these considerations in mind, preservation of T-cell function suggests that the discarded NT fractions could be considered as an alternative product for DLI, saving the donor the cost and the discomfort of a new apheretic session. In particular, in case a second Aph cannot be performed for whatever reason, NT cells represent a feasible alternative.

A mean number of 10^10^ αβ T cells were present in the NT fractions. This number largely exceeds the number of T cells theoretically encompassing a full human repertoire (74, 75). In particular, memory T cells, which represent the subset most relevant for protection from opportunistic infections, is represented by approximately 10^6^ different clonotypes with a frequency of each pathogen-specific T cell (including CD4 and CD8 cells) ranging between 0.05 and 5% in peripheral blood (76). An alternative estimate has been proposed by assessing the diversity of the TCR β chains only by high throughput sequencing (74). In this case, the diversity of the memory repertoire was calculated at around 10^6^ different clonotypes, but, considering combinatorial diversity contributed by TCR α chains, the figure can be remarkably higher. These evaluations imply that each clonal specificity is represented by at least one T cell in a sample containing a few million T cells. Therefore, the discarded NT fractions contain thousands of cells from each memory T-cell clone specific for pathogen antigens, redundantly reflecting scores of replicates of the same memory repertoire. Even if far from being accurate, these figures underline how valuable the NT fractions can be from an immunological point of view.

With respect to the observation that specific responses were occasionally higher in NT cells than in Aph cells (or in NT plus CB cells), we should keep in mind that the simplest functional unit for cellular immune response is represented by one specific T cell interacting with one APC. Other neighboring cells may interfere with this interaction, and a third party may not be welcome. Since NT fractions are enriched in T and B cells, antigen-driven cellular interactions may be facilitated. This may account for better responses in NT fractions than in Aph samples, or than in NT fractions supplemented with CB cells. CB fractions, in fact, in addition to MoDC, contain other cell types, such as NK cells and γδ T cells, which may physically interfere with antigen-driven T–B interactions. On the contrary, it is hard to explain why we occasionally observed specific responses higher in Aph than in NT samples. In this case, it is possible that the column elution procedure for NT fractions, which follows the collection of CB fractions to be transplanted, might occasionally affect T-cell functions in NT samples. The observed variability suggests that T-cell functions should be checked in each preparation.

In case large numbers of T cells are required, an aliquot from the mobilized apheretic donation (48) may not suffice. On the other side, a second Aph is hard to propose in case a matched unrelated donor is used for the allograft, due to donor or registry denial, or it may sometimes not be feasible in the haploidentical donor setting for personal or health reasons. Altogether, these considerations make the use of NT cells particularly appealing.

**Figure 6:**
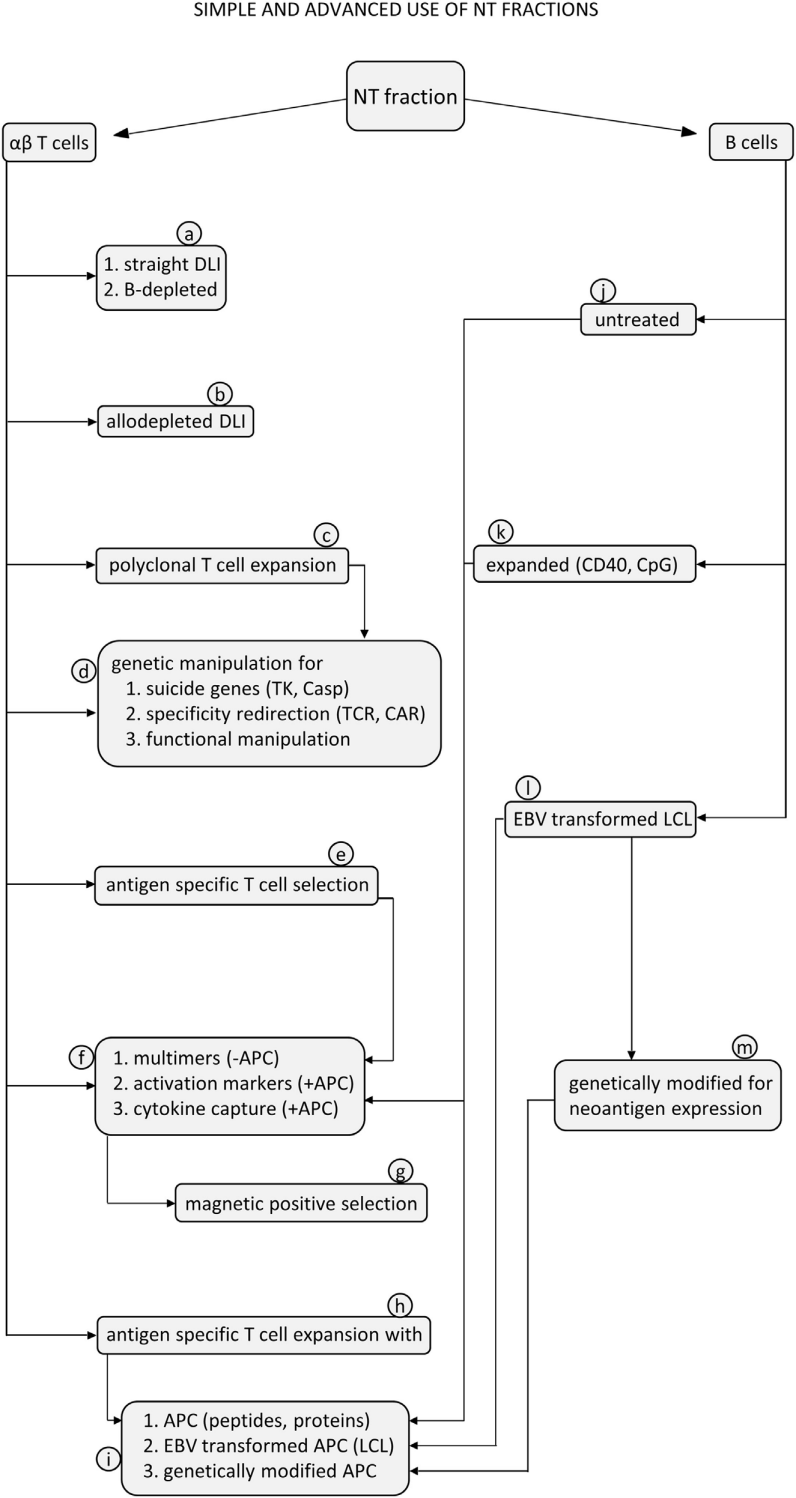
**Flow diagram for proposed exploitation of αβ T cells and B cells from non-target (NT) samples using simple or advanced procedures**. T cells contained in the NT fractions can be used for unmanipulated donor lymphocyte infusion (DLI) (a1) or after B cells have been removed (a2). This can be achieved by cell preincubation to allow internalization and degradation of the paramagnetic beads, followed by a depletion step with anti-CD19 beads only. T cells can be manipulated to reduce the frequency of alloreactive cells (b) for graft-versus-host disease prevention by using one of the methods already proposed in previous studies, as recently reviewed (75). T cells can be polyclonally expanded with anti-CD3 antibodies (c) (33) to produce large numbers of activated cells ready for subsequent genetic manipulations, such as introduction of suicide genes, like thymidine kinase (TK) or inducible caspase-9 (iCasp9) (d1) (32, 79), for redirection of specificity by introducing a chimeric antigen receptor (d2) (35) or for functional manipulation by adding genes for accessory molecules or cytokines (d3) (35, 80). T cells can be selected based on specificity for antigens of relevant pathogens (e) by using several strategies (f). Multimer selection does not require previous antigen stimulation in the presence of APC (f1), while selection based on expression of activation markers (f2) or on cytokine capture (f3) does require antigen activation with APC. APC can be untreated autologous B cells (j), expanded (k), or modified B cells (l, m). These procedures are followed by positive selection of activated T cells usually based on paramagnetic microbeads (g) to select T cells specific for adenovirus (67), CMV (81), or EBV (68). Finally, T cells can be stimulated and expanded *in vitro* to generate established T cell lines specific for antigens of relevant pathogens, using different protocols (h) (31). As an example, peptides and proteins can be used in the presence of APC as autologous B cells (i1). EBV-transformed autologous B cells can be used to activate EBV-specific T cells (i2). Genetically modified APC can express antigens of different viruses (i3) (82) to obtain multi-virus-specific T-cell lines. On the other side, B cells can be considered adequate APC under different conditions. B cells can be used untreated (j) or simply irradiated, terminally expanded *in vitro* with CpG (83), or long-term expanded with anti-CD40 antibodies (k) (84, 85). B cells can be transformed with EBV to generate lymphoblastoid cell lines (LCL) (l), which can directly present EBV antigens to T cells or can be genetically modified to express new viral antigens (m) (82).

Our view on recovered NT cells is not limited to their exploitation as a substitute for DLI. Thanks to their intact functions, these cells can be candidates for additional *in vitro* manipulation before reinfusion. A note of caution should be introduced, though, considering that GCSF has been recently shown to affect some functions of CD8 T cells, without impairing their *in vitro* expansion (77, 78). In this regard, it will be interesting to test whether this effect is reversible upon *in vitro* culture. Cell manipulation may include specific allodepletion, selection, and expansion of specific T cells, genetic manipulations to introduce suicide genes, or novel chimeric receptors for their retargeting. These possibilities, which need to be experimentally validated on NT cells, are outlined in Figure 6, with relevant references provided in the legend.

With respect to B cells present in the NT fractions, in addition to their intact APC function demonstrated here, three functional features are being tested in experiments in progress, as illustrated in Figure 6. The first one is *in vitro* expansion with CpG or with anti-CD40 antibodies in order to produce large numbers of APC to be used in restimulation cycles for the generation and expansion of antigen-specific T-cell lines. The second one is related to EBV transformation of these B cells available in large numbers for production of lymphoblastoid cell lines (LCL) to be used for generation of EBV-specific CTL (86, 87) using a faster approach as compared with direct LCL generation from peripheral blood samples. This may permit generation of EBV-specific CTL lines with a procedure swifter than advocated in standard protocols. The third one relates to B-cell removal from the T-cell products. In fact, if multimers are used for T-cell selection on a magnetic column right after the αβ T-cell/B-cell depletion procedure, B cells, which are still coated with anti-CD19 magnetic beads, might be retained by the magnetic column and copurify together with multimer-labeled, antigen-specific T cells. Therefore, a preincubation step is required to allow internalization and degradation of the magnetic beads used for B-cell depletion. In case specific T cells are selected with other methods, based on the expression of activation markers (76) or on the cytokine capture system (62, 63), prior antigen stimulation, which depends on the presenting function of B cells, is needed. In this case, beads used for the first depletion are internalized and degraded during the incubation phase and do not interfere with the second depletion phase. Since specific T cells are positively selected, B cells are excluded from the final product. On the other side, if T cells are stimulated and repeatedly expanded to produce established T-cell lines, B cells present among the cells used as APC can be physically removed by negative selection with anti-CD19 beads or can be neutralized by previous irradiation.

In conclusion, our data are in agreement with other reports testing the possible recovery of cellular products obtained after manipulation procedures (48–51, 88–90), suggesting that fractions comprising αβ T cells and B cells obtained as by-products of *ex vivo* manipulation (38) of apheretic or bone marrow grafts are a valuable source of cells, which may be useful for adoptive immunotherapy after HSCT.

## Ethics Statement

The work described in this study was approved by the institutional Ethics Committee, IRCCS Bambino Gesù Children’s Hospital, Rome, Italy (approval #938/LB), and a written informed consent was obtained from the donors.

## Author Contributions

GLP, SDC, MM, and FL conceived and designed the experiments. GLP, SDC, SB, EG, EC, VB, CQ, and IC performed the experiments. BL, PM, DP, LB, and AB selected the donors and contributed with ideas for experimental design and performance. GLP, SDC, and FL wrote the manuscript with edits from AB and MM. All the authors revised and approved the contents of the manuscript.

## Conflict of Interest Statement

The authors declare that the research was conducted in the absence of any commercial or financial relationships that could be construed as a potential conflict of interest.
